# Evaluation of the Clinical Impact of ISO 4049 in Comparison with Miniflexural Test on Mechanical Performances of Resin Based Composite

**DOI:** 10.1155/2015/149798

**Published:** 2015-02-28

**Authors:** Luigi Calabrese, Francesca Fabiano, Lucio Maria Bonaccorsi, Valerio Fabiano, Chiara Borsellino

**Affiliations:** ^1^Department of Electronic Engineering, Chemistry and Industrial Engineering, University of Messina, Contrada di Dio, 98166 Messina, Italy; ^2^Department of Experimental, Specialized Medical-Surgical and Odontostomatological Sciences, University of Messina, Via Consolare Valeria 1, 98125 Messina, Italy; ^3^Department of Civil Engineering, Computing, Construction, Environmental and Applied Mathematics, University of Messina, Contrada di Dio, 98166 Messina, Italy

## Abstract

The aim of this study was to evaluate the effect of different specimens dimensions on the mechanical properties of a commercial microfilled resin composite by using a modified ISO 4049 standard protocol, that generally provides specimen dimensions of 25 mm length × 2 mm width × 2 mm height; these standard dimensions are not clinically realistic considering the teeth diameter and length average. Furthermore, the overlapping irradiations required lead to specimens that are not homogeneous with the presence of some flaws due to packaging steps. For this reason, a miniflexural test was employed in this work both to simulate clinically realistic dimensions and to concentrate fewer defects. The flexural tests were performed at varying span length, in the range between 18.5 mm as stated by the ISO 4049 flexural test (IFT) and 10.5 mm according to the miniflexural test (MFT), at the increasing of layers with a 1 mm buildup multilayering technique. The results evidenced the impact of specimen dimensions on mechanical performances and consequently stability of resin-based composite with the formation of an asymmetrical structure which possesses higher stiffness and strength at increasing layering steps.

## 1. Introduction

Dental resin-based composites are widely used in restorative dentistry since they have been introduced for the first time in the middle of 1960 [1–5]. Compared to dental amalgams, they have less safety concerns, have simple usability, and possess better aesthetic properties [6, 7]. Nevertheless, amalgam still performs better mechanical properties than composite.

One of the laboratorial tests widely used to evaluate the mechanical behavior of resin composites is flexural strength test according to the International Standard ISO 4049. Modifications of test parameters are commonly reported in the literature due to the possibility to increase their reproducibility and clinical relevance in oral environment. However, the results obtained from the comparison between different methodologies were shown to be inconsistent, especially when different specimen dimensions are considered [8–10].

For this reason an accurate experimental parameters selection is required in order to analyze the effect on strength related properties which is commonly considered a reliable clinical performance indicator for dental composites.

By analyzing the parameters that affect physical, mechanical, and chemical properties of filled composites we should focus on some important concepts.

The flexural properties, like flexural strength and modulus, of composites are strongly influenced by the *L*/*h* ratio (span length/thickness); the ratio can be varied either by changing the support span at constant thickness or by changing the specimens thickness at constant span [8]. This was assumed to be due to shear stress contribution inside the specimen instead of tensile stress which lowers *L*/*h* ratios [10].

In addition, adequate polymerization of composite restorative materials is fundamental for obtaining optimal ideal clinical performances. Low conversion rates, in fact, affect several important parameters such as flexural strength, fatigue, solubility, discoloration, and biocompatibility, thus limiting the lifespan of the composites [11–14].

The large bar-shaped specimens, as stipulated in ISO 4049, are difficult to prepare without flaws especially with compule packaging. Furthermore, several overlapping irradiations [240*s* (40*s* × 3 × 2)] are required as the exit window of all clinical light-cure units is smaller than 25 mm. This leads in specimens that are cured inhomogenously. Therefore the resulting polymerization shrinkage of resin composites causes the presence of residual stresses within regions of incomplete polymerization. Consequently, the overlapping curing regime of three-point flexure bar-shaped can affect the reliability of fracture strength data compared with specimens fabricated using a more clinical relevant protocol [9].

Specimen dimensions, curing time, and layering procedure are fundamental parameters that influence the resin catalysis and consequently the mechanical properties of the final product [15–17]. For this reason, the aim of this study was to better clarify the controversial information of the scientific community on the mechanical properties of microfilled composites at varying specimen dimensions (length *L*: 12 mm/25 mm, width *w*: 2 mm, and thickness *s*: 1.0, 1.0 + 1.0, and 1.0 + 1.0 + 1.0 mm). In particular the three-point bending test was performed, respectively, at 18.5 mm (IFT) and 10.5 mm (MFT) span length, through an 1 mm incremental technique.

## 2. Materials and Methods

A commercial light-curing resin (Quadrant Universal LC, supplied by Cavex Holland BV) was chosen for the tests. It was a Bis-GMA resin loaded with well-dispersed fillers (Ba-Al-F-silicate glass, size 0.02–2.00 *μ*m, and dispersed colloidal silica, size from 0.02 to 0.07 *μ*m and mean particle size about 0.045 *μ*m). This universal microhybrid composite was utilized because of its general use in most anterior and posterior restorations based on its combination of strength and polishability.

A customized stainless steel mold was filled with the uncured composite paste, shade A2; hence, the two sides of the mold were placed between two glass slides with a polyester film interposed between the glass and the mold. With a 13 mm diameter light cure tip the polymerization for 12 × 2 × 1 specimens group can be achieved with a nonoverlapping light-curing procedure (20*s* × 2) while for 25 × 2 × 1 group overlapping light-curing exposure is required (20*s* × 3 × 2). For this reason, in the 25 × 2 × 1 group, after the glass slide was removed, the exit window of the visible light unit (Optilux-501, Kerr, CT, USA) was positioned at the center of the specimen and against the glass slide and then the specimen was irradiated for a polymerization time of 20 s (as recommended by the manufacturers). After the photoactivation of the specimen's center, the exit window was moved to the section next to the center, overlapping the previous section by half the diameter of the exit window; the same goes for the section on the other side of the center; both sides were irradiated as recommended by the manufacturer.

Therefore, the specimens were stored in distilled water at 37°C for 24 h before the testing.

Preliminary physical parameters, obtained according to ASTM D 3171, are reported in Table 1.

The percentage of voids confirms that the sample preparation was performed correctly.

Three-point bending test was performed using ISO 4049 flexural test (IFT) and a miniflexural test (MFT). The test was performed at room temperature using a universal testing machine (Tenso Test TT2, 5-GU, Lonos Test, Monza, Italy) with a 10 N and 2 kN load-cell with 0.001 N sensitivity. The cross-head speed was 0.75 mm/min. The test was replicated 10 times to better identify statically the mechanical performances. The flexural properties were measured using rectangular bar-shaped samples (length *L* = 12–25 mm, width *w* = 2 mm, and thickness *s* = 1.0–2.0–3.0 mm). The test was replicated 10 times for the two experimental groups to better identify statically the mechanical performances.

The strength and stiffness were determined, respectively, by using the following equations:

(1) σf3·P·L2·b·h2,

(2) Ef=L348·I·PΔ,

where *σ*_*f*_ is the flexural stress and *E*_*f*_ is the apparent modulus of elasticity obtained by flexural test. *L* is the span length, *h* and *b* are, respectively, height and width of beam. *I* is the moment of inertia and *P*/Δ is the slope of load-deflection data.

Furthermore the three-point bending test was used, according to ASTM D198, to determine the shear modulus of the restorative composite.

During the three-point bending test, a certain extent of displacement measured was dependent on the shear resistance that characterizes the material investigated. This correlation was related to the ratio between the distance of the supports (*L*) and the thickness of the sample (*h*). Higher shear stresses intervene when the *L*/*h* ratio is low; on the contrary increasing this ratio becomes irrrelevant. By carrying out several three-point bending tests, which differ in the ratio of *L*/*h* (with *h* constant and *L* variable), and by measuring the apparent elastic modulus, it was possible to separate Young's modulus (*E*) from the shear modulus (*G*). Consequently, this standard specifies that a beam is tested via center-point loading over multiple spans per specimen. Within this procedure, the apparent modulus of elasticity was determined at multiple aspect ratios.

The true modulus of elasticity differs from the apparent modulus of elasticity because it neglects deflection due to shear. In order to determine the true moduli of elasticity, a linear interpolation of the apparent moduli of elasticity and the aspect ratios at which they were measured is requested. The resulting intercept is the inverse of the true modulus of elasticity. The inverse slope of the interpolating straight line, modified by a shape factor, is the shear modulus. The equation used to relate these parameters is the following:

(3) $$\begin{array}{c} \frac{1}{E_{f}}=\frac{1}{E}+\frac{1}{K\cdot G}\cdot {\left(\frac{h}{L}\right)}^{2}, \end{array}$$

where *E* is the true modulus of elasticity, *G* is the modulus of rigidity, and *K* is a shape factor.

Afterward microhardness tests were carried out along the cross section of each sample. Microhardness tests were performed by using a Future-Tech Microhardness Tester FM-300 (Vickers indenter and compressive load 100 g). 100 replicas of test were carried out. The degree of conversion is defined as the percentage of reacted C=C double bonds [12]. Hardness has been shown to be a good indicator of conversion of double bonds and was therefore used in the present study as an indirect measurement of conversion. Polymerization of resin-based composites leads to a highly cross-linked structure, but both steric hindrance and vitrification cause residual unsaturation by pendant methacrylate groups and free monomers. This not fully cured external layer is essential for the chemical bond of further layers. This layer, generally, is removed through polishing fine diamonds, flexible discs, and silicon polishers after clinical composite restoration [18]. For this reason, before performing the microhardness test, this layer was removed by lapping.

## 3. Results and Discussion 

### 3.1. Flexural Tests

With the purpose to better clarify the effect of 1 mm layering buildup technique, with a practical 20 s curing time, on the mechanical performances of a microfilled composite, the flexural tests were performed at varying span length, in the range between 18.5 mm (IFT) and 10.5 mm (MFT). In particular, a miniflexural test was employed to simulate a clinically realistic specimen dimensions whereas the mesiodistal diameter of molars is about 11 mm and the cervicoincisal length of central incisors is around 13 mm.

Under flexural condition, when the load is applied, the bar specimen bends. The principal stresses on the top surfaces are compressive, whereas those on the lower surfaces are tensile. While the tensile and compressive moduli are generally the same for metals, they may differ significantly for polymeric composites. The advantage of flexural modulus for polymeric composites is that it describes the combined effects of compressive and tensile deformation [19].

**Figure 1:**
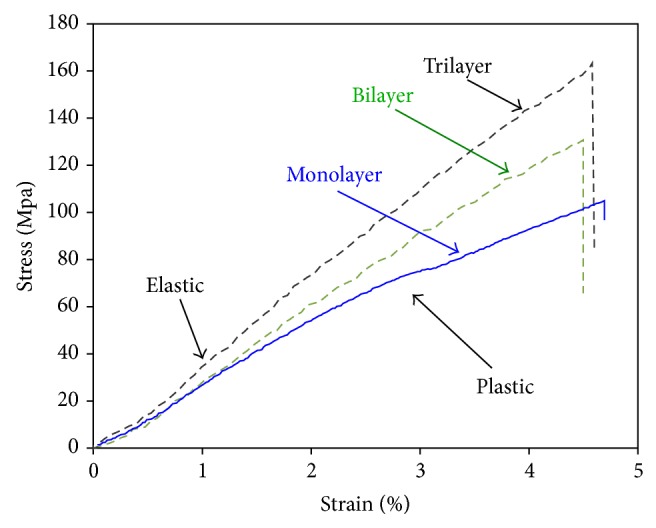
Stress-strain curves of samples at different layering for 18.5 mm span length.

Figure 1 shows the evolution of stress-strain curve at increasing layering steps for the three-point flexural test configuration with 18.5 span length.

The analysis of stress-strain pattern evidenced that the mechanical properties of the composite resin increase at each layering step. In particular the monolayer sample was able to carry low stresses with high strains due to its significant plastic behavior. In fact, it could reach high deformation (the maximum deflection for some samples was quite double of the thickness) without the insurgence of critical failure conditions, even if the maximum stresses were relatively low.

The neutral axis of a beam curve, which was bent upwards in the device, was moved slightly from the centroid towards the center of curvature, giving a nonlinear distribution of the stresses through the thickness of the beam. For small values of thickness, the beam displacement of the neutral axis was very small and the disturbance to distribution ratio of linear stress was assumed to be negligible [20].

If the maximum deflection was not small compared to the beam depth, linear beam theory cannot be employed without an error. West [21] examined large deflections of three-point loaded beams, and from such results a definitive ratio of beam length-to-depth (*L*/*d*) ratio can be determined for valid application of simple beam formulas. Based on its considerations, the error for beams with large deflection under three-point flexural test is under 5% up to *L*/*d* ratio of 250 [22]. Furthermore, based on Roark approach [23], the error due to low curvature radius can be estimated under 2% [24]. Consequently, for our fixture configuration, the data of the analysis differed slightly from the Euler-Bernoulli theory (small deflections of a beam) and (1) and (2) could be used to compare the mechanical performances of the composite paste.

The monolayer sample showed a nonlinear curve with a first elastic region at low deformation. Afterward, at increasing deflections, the stress-strain curve exhibits a progressive deviation from linear trend. At about strain 1.5%, a transition between elastic to plastic regime could be discriminated. This behavior could be justified considering that the photoactivable resin was not full cured. The unreacted compounds, characterized by a high mobility, influence significantly the plastic deformation inducing low strength and stiffness on the sample. Instead for high layering steps, the stress-strain curve became quite linear. These samples maintained an elastic regime also at high strain, evidencing an elastic-brittle behavior typical for well cross-linked composite resin [25]. Anyway, all samples have highlighted a quite similar failure strain.

The degree of conversion has a substantial effect on final mechanical properties. In fact, during polymerization, dental resin composites transform from plastic viscous through a rubbery viscoelastic into an elastic glassy stage. During and after the vitrification stage, the major rate of shrinkage stress occurs, due to residual stresses within regions of incomplete polymerization, specially under overlapping irradiations. In fact, to compensate for the polymerization of the central region of the 25 × 1 × 2 rectangular specimen, the associated postgel shrinkage stresses were accommodated by deformation of the adjacent, partially irradiated regions. As the adjacent regions of the bar-shaped specimen were irradiated, the polymerization shrinkage stress of the overlapping areas of resin composite placed the cured central portion under tensile stresses [9]. After the gel-point, steric hindrance becomes prominent and the elastic properties are measurable. The elastic modulus increases with increasing conversion reaching its final level at the glassy stage [14]. If the deepest layers of composite restorations are not adequately cured, the elastic modulus at the bottom will be lower than that at the surface; this can increase the material strain under masticatory forces.

During the flexural test, the stresses change direction within the specimen between the top and bottom surfaces, with both stress and strain being zero at the region of change (neutral axis) [19]. Shear stress is also produced near the supported ends of the specimens but does not play a significant role in the fracture process when the distance between supports is large (i.e., IFT).

**Figure 2:**
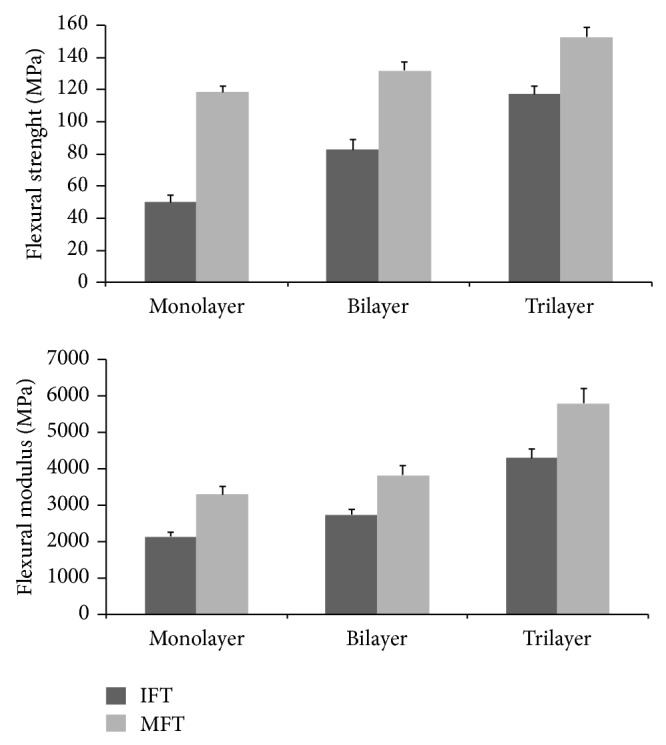
Comparison of flexural strengths and modulus (Mpa) with IFT and MFT.

Averages of flexural strength and modulus values of the two experimental groups are reported in Figure 2.

The bending strength and modulus increase with the steps of layering both in IFT compared to those in MFT. The difference was statistically significant for flexural strength and Young's modulus values in monolayer samples of IFT group in comparison with the monolayers in MFT group (*P* < 0.01).

For bilayers as well as trilayers higher flexural strength and Young's modulus values were observed in IFT (*P* < 0.01).

When the distance between supports is smaller (i.e., MFT), flexural properties observed express the combined effects of compressive, tensile, and shear deformation that could induce a premature fracture of the sample. Nevertheless, higher specimen dimensions (e.g., IFT samples) could evidence cross-linking heterogeneity due to overlapping curing procedure. This leads to residual stresses within region of incomplete polymerization that influence the mechanical performances of the composite paste. On the contrary smaller specimens are more subject to a homogeneous curing distribution than to less defects content [19, 26].

With the purpose to minimize this effect, the sample's irradiation steps were carried out taking into account the distribution of the overlapping areas. Furthermore, as described in the following paragraph, we proposed 1 mm layering procedure to obtain a homogenous polymerization between top and bottom surfaces of the sample [27].

This may explain the generally significant increase of the elastic modulus and flexural strength observed with IFT compared with MFT.

**Figure 3:**
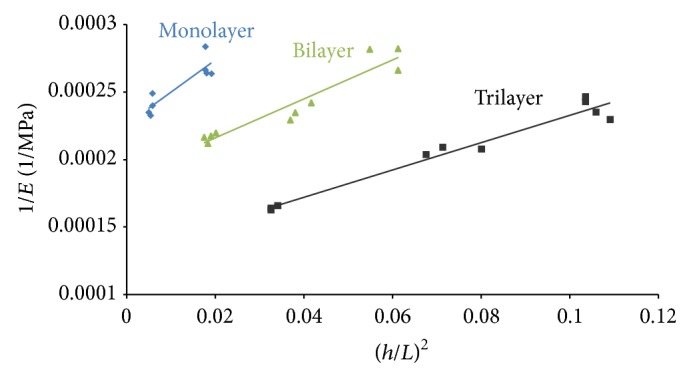
Trends comparison of 1/*E*_*f*_ in terms of (*h*/*L*)^2^ for monolayer, bilayer, and trilayer samples.

In particular, it was confirmed that, by increasing the span length from *L* = 10.5 to *L* = 18.5 mm, the apparent elastic modulus *E*_*f*_ increases. Instead a slight reduction in the flexural strength was observed for MFT samples compared with IFT ones. This is due to the enhancement of shear strain addition (angular sliding) compared to flexural strength contribution when the distance between the support was reduced. To obtain the effective elastic modulus of this dental material, as value independent from span length, an extrapolation technique was used which allowed to obtain the Young's modulus, by eliminating the shear deformation contribute from the apparent modulus *E*_*f*_, by using the procedure reported in the experimental part. Analogously, by the slope of interpolation line, it was possible to determine the shear modulus. The flexural data are reported in Figure 3.

The trilayer samples evidenced the highest apparent elastic modulus, *E*, (consequently the lowest 1/*E* contribution) in all range of spans. Reducing the layering steps, the apparent elastic modulus progressively decreases. Each sample group had evidenced a quite linear trend on the graph. Interpolating by a linear curve it was possible to extrapolate the elastic and shear stiffness of each specimens group, obtaining a detailed mechanical characterization of the composite paste. The results are summarized in Table 2.

Increasing the number of layers, during the restoration phase, can lead to an enhancement of both elastic and shear stiffness. An elastic modulus of about 4400 Mpa for 1 mm monolayer was obtained. Instead we observe an elastic modulus for trilayer samples about 70% higher than monolayer one. These considerations are confirmed by analyzing the shear modulus results. The shear modulus observed for trilayer samples is two times higher than the monolayer one. The mechanical performances of monolayer and bilayer were slightly similar (anyway a significant difference of *G* modulus was observed).

This behaviour plays a main role on the interlaminar stresses, favoring a better interfacial mechanical stability on samples with more high and stable performances. Consequently samples obtained with a 1 mm multilayering procedure could be able to provide a better adhesion and interlaminar shear performances.

The combined knowledge of the longitudinal and shear elastic properties of resin composites is essential for their correct use in restorative dentistry. In fact, in the oral environment, the dimensions are compatible with the MFT, which showed values of stiffness and strength lower than the IFT ones. Consequently an incorrect evaluation of these parameters can lead to marginal breakdown or fracture of the bulk of the restorations which are subjected to considerable stress-bearing (Classes I, II, and IV restorations) where a high flexural strength is required to withstand biting forces without fracture and also a high modulus or stiffness is necessary during the restoration to maintain its shape under load.

A combined IFT-MFT testing approach can be able to overcome these deficiencies by providing useful information for the prediction of the mechanical behavior of resin composites under realistic conditions of application.

### 3.2. Microhardness Tests

**Figure 4:**
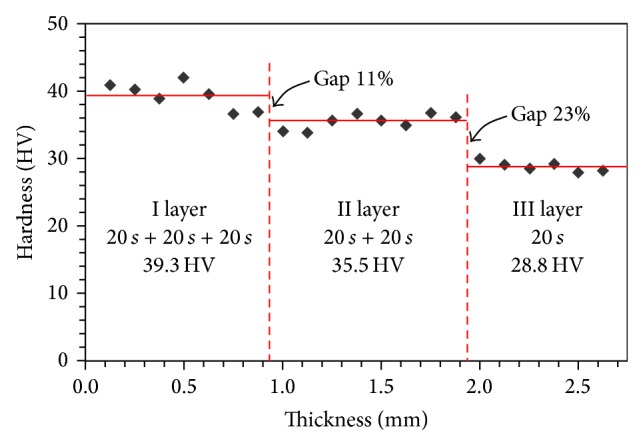
Microhardness HV along the thickness of a trilayer reference sample.

With the purpose to evaluate the influence of layering steps on the hardness properties microhardness measurements were carried out along the cross section of the composite samples. The microhardness value can be considered an affordable parameter to relate with the level of curing of the composite paste [28, 29]. In this way, the cross-linking conversion level can be related with the geometrical thickness of the layers. The results for a trilayer sample are reported in Figure 4.

The figure shows how the gap between the layers with a time of cure of 20 sec and 40 sec is more marked (23%) than the gap between the layers cured in 40–60 sec (11%). Due the multistep layering procedure, the sample exhibits an asymmetrical structure, with a very hard thick region with high microhardness values and a soft superficial region. The latter region, characterized by only 20 s of curing time, has a microhardness value about 40% lower than the hardest layer, characterized by a 60 sec of curing time. This intrinsic anisotropy influences the stress distribution and could increase the interlaminar stresses, favoring debonding or delamination [30] of the restored tooth. This leads to the conclusion that to reduce the differences in HV values and therefore the mechanical properties between the various composite laminae it is necessary either to perform layers of 1 mm thick maintaining the standard cure time of 20 sec, or to raise the layers thickness and consequently the curing time. However, considering that as the thickness of the composite increases, the number of photons available to raise CQ to the activated state is limited by absorption and scattering factors associated with the overlying resin. This reduces the probability of collision of CQ with an amine [12]. An optimal procedure might be to implement a multistep layering phase with the purpose to minimize the mechanical performances discrepancies between the layers and finally to apply a postcuring treatment with the purpose to homogenize the superficial layer with the underlying ones. Similar results were obtained in a number of works present in literature that have addressed the problem of incremental technique, such as Kwon et al. that show how cuspal deflection caused by polymerization shrinkage may be reduced by a 1 mm incremental filling technique in order to obtain a long restoration lifespan [31].

This information may be useful in order to provide a correct procedure of deposition and resin composite curing during dental reconstruction. From the dental point of view, the mechanical behavior of the reconstructed tooth is the result of the combination of the performance of several layers, characterized by different hardness and stiffness. In this sense, a more detailed research will be developed to verify the stress distribution for a dental multilayer system in order to be able to predict the final performance of a real reconstructed tooth.

## 4. Conclusions

The experimental results evidenced that the ISO 4049 flexural test in comparison with miniflexural test limits the physical properties extrapolation of resin composites. In order to provide realistic clinical conditions, our results show how separate the flexural properties are from the shear stress, by carrying out several three-point bending tests, which differ in the ratio of *L*/*h* (span length/thickness) and in the incremental technique of specimens.

Furthermore, the layering procedure, necessary to realize a dental reconstruction, played a key role in the mechanical performances of resin composite. Best mechanical performances were observed for trilayer samples.

These last trilayer samples evidenced elastic and shear modulus, respectively, two and four times higher than monolayer ones. Anyway this configuration evidenced an anisotropic mechanical behavior with a thick hard layer (the first and second depositions have quite similar mechanical performances) and a soft external layer (last deposited layer evidenced very low performances compared with other ones). This last is characterized by a hardness value about 40% lower than the hardest one. To reduce this discrepancy a postcuring step after the multilayering procedure was proposed.

## Figures and Tables

**Table 1:** Physical properties of the composite sample.

Filler vol %	56.7
Filler wt %	68.0
Density [g/cm^3^]	2.075
Voids [%]	2.42

**Table 2:** Elastic and shear modulus of multilayer resin composite samples.

	*E* [MPa]	*G* [MPa]
Monolayer	4416.1	497.7
Bilayer	5346.3	813.4
Trilayer	7591.8	1166.0
